# Aβ‐Induced Modulation of miRNA‐212 in Cerebral Endothelial Cells

**DOI:** 10.1002/alz70855_106447

**Published:** 2025-12-24

**Authors:** Micaelly Alves, Silvia Fossati

**Affiliations:** ^1^ Alzheimer Center at Lewis Katz School of Medicine, Temple University, Phialdelphia, PA, USA; ^2^ Temple University, Phialdelphia, PA, USA; ^3^ Alzheimer's Center at Temple, Lewis Katz School of Medicine, Philadelphia, PA, USA

## Abstract

**Background:**

Cerebral vascular endothelial cells (CVECs) play a critical role in maintaining blood‐brain barrier (BBB) integrity and cerebral homeostasis. Dysfunction of the cells composing the BBB is an early hallmark of Alzheimer's disease (AD) and is associated with amyloid beta (Aβ) deposition. The accumulation of Aβ around cerebral blood vessels characterizes Cerebral Amyloid Angiopathy (CAA), which is a co‐pathology present in over 80% of AD patients. Our lab has previously demonstrated that Aβ alters BBB composition, increases barrier permeability and induces CVEC apoptosis. The miR‐212/132 family has been shown to be neuroprotective and to be reduced in neurodegenerative diseases, including AD. While the specific targets of these miRNAs have been explored in neurons, their contributions to vascular health in AD and CAA remains unexplored.

**Method:**

We investigate the hypothesis that Aβ promotes endothelial cell dysfunction via modulation of miRNA‐212. We evaluated changes in gene expression of miR‐212 and miR132 via RT‐qPCR in CVEC challenged with pathological Aβ species: Aβ40‐E22Q, the Dutch mutant strongly associated with CAA pathology, as well as Aβ42. Moreover, by measuring trans‐endothelial electrical resistance (TEER) and tight junction proteins and mRNA levels, we assessed whether the negative effects of Aβ on BBB stability could be rescued by restoring normal miR‐212 levels.

**Result:**

Our results show a time‐dependent biphasic modulation of miR‐212 in CVEC, which can be correlated with the progression of Aβ burden. Further, we determined how BBB and CVEC impairment caused by Aβ pathology are affected by miR‐212 mimics and inhibitors.

**Conclusion:**

Aβ induces modulation of miR‐212 over time, causing dysregulation in CVEC function, potentially reversible by miRNA therapeutic approaches.

